# Metabolic Changes in Early-Stage Non–Small Cell Lung Cancer Patients after Surgical Resection

**DOI:** 10.3390/cancers13123012

**Published:** 2021-06-16

**Authors:** Naseer Ahmed, Biniam Kidane, Le Wang, Zoann Nugent, Nataliya Moldovan, April McElrea, Shiva Shariati-Ievari, Gefei Qing, Lawrence Tan, Gordon Buduhan, Sadeesh K. Srinathan, Michel Aliani

**Affiliations:** 1 Research Institute in Oncology and Hematology, CancerCare Manitoba, Winnipeg, MB R3E 0V9, Canada; bkidane@hsc.mb.ca; 2 Department of Radiology, Section of Radiation Oncology, Rady Faculty of Health Sciences, University of Manitoba, Winnipeg, MB R3T 2N2, Canada; nmoldovan@cancercare.mb.ca; 3 Department of Community Health Sciences, Rady Faculty of Health Sciences, University of Manitoba, Winnipeg, MB R3E 0W3, Canada; 4 Department of Surgery, Rady Faculty of Health Sciences, University of Manitoba, Winnipeg, MB R3A 1R9, Canada; LTan@hsc.mb.ca (L.T.); gbuduhan@hsc.mb.ca (G.B.); ssrinathan@hsc.mb.ca (S.K.S.); 5 CancerCare Manitoba, Winnipeg, MB R3E 0V9, Canada; wangl319@myumanitoba.ca (L.W.); znugent@cancercare.mb.ca (Z.N.); 6 St. Boniface Hospital Albrechtsen Research Centre, Winnipeg, MB R2H 2A6, Canada; AMcElrea@sbrc.ca (A.M.); SShariati@sbrc.ca (S.S.-I.); 7 Department of Pathology, Rady Faculty of Health Sciences, University of Manitoba, Winnipeg, MB R3E 3P5, Canada; GQing@sharedhealthmb.ca; 8 Department of Food and Human Nutritional Sciences, University of Manitoba, Winnipeg, MB R3T 2N2, Canada

**Keywords:** lung cancer, surgical resection, metabolomics, biofluids

## Abstract

**Simple Summary:**

Considerable progress in the treatment of non–small cell lung cancer (NSCLC) has been made possible by large-scale technologies that scan the gene expression in tumor cells. While gene expression is informative, it is the changes to cellular metabolism that directly affect the initiation and the progression of the disease. Altered metabolic processes in cancer include how the tumor cells handle fat, proteins, and sugar, produce energy, divide (grow), or migrate. We have used nuclear magnetic resonance and mass spectrometry to survey and document the metabolic changes in blood and urine samples collected from NSCLC patients before and after their lung tumors were surgically removed. We found several molecular compounds that changed in abundance in the blood or urine after surgery, many of which are related to cancer cell metabolism. Further documentation of these changes in large patient populations will lead to non-invasive ways to screen, diagnose, or monitor disease progression in lung cancer patients.

**Abstract:**

Metabolic alterations in malignant cells play a vital role in tumor initiation, proliferation, and metastasis. Biofluids from patients with non–small cell lung cancer (NSCLC) harbor metabolic biomarkers with potential clinical applications. In this study, we assessed the changes in the metabolic profile of patients with early-stage NSCLC using mass spectrometry and nuclear magnetic resonance spectroscopy before and after surgical resection. A single cohort of 35 patients provided a total of 29 and 32 pairs of urine and serum samples, respectively, pre-and post-surgery. We identified a profile of 48 metabolites that were significantly different pre- and post-surgery: 17 in urine and 31 in serum. A higher proportion of metabolites were upregulated than downregulated post-surgery (*p* < 0.01); however, the median fold change (FC) was higher for downregulated than upregulated metabolites (*p* < 0.05). Purines/pyrimidines and proteins had a larger dysregulation than other classes of metabolites (*p* < 0.05 for each class). Several of the dysregulated metabolites have been previously associated with cancer, including leucyl proline, asymmetric dimethylarginine, isopentenyladenine, fumaric acid (all downregulated post-surgery), as well as N6-methyladenosine and several deoxycholic acid moieties, which were upregulated post-surgery. This study establishes metabolomic analysis of biofluids as a path to non-invasive diagnostics, screening, and monitoring in NSCLC.

## 1. Introduction

There has been considerable progress in our understanding of lung cancer biology in the last two decades. Checkpoint inhibitors against the programmed death ligand-1 (PD-L1) as a primary treatment or in combination with chemotherapy have changed the landscape of non–small cell lung cancer (NSCLC) with significantly improved outcomes [1]. Several actionable driver mutations, namely epidermal growth factor receptor (EGFR), anaplastic lymphoma kinase (ALK), and c-ros oncogene 1kinase (ROS1), have been identified using next-generation sequencing techniques, resulting in specifically targeted drug therapy and improved outcomes in patients harboring these mutations [2,3,4]. While it is true that genomics, transcriptomics, and proteomics may predict the biological behavior of malignant cells, it is their metabolic alterations that play a direct and vital role in tumor initiation, proliferation, and metastasis. Some examples of metabolic alterations that are critical to the survival and proliferation of malignant cells include accelerated glycolysis, the formation of lactic acid, changes in the citric acid cycle, amino acid metabolism, and cell membrane synthesis [5,6,7,8,9]. Metabolomics research therefore offers a means to observe the current status of the cellular microenvironment. Recently, there has been movement to develop and incorporate metabolomics in NSCLC research to uncover the biomarkers for applications such as screening, pathogenesis, histopathological classification, and possible therapeutic interventions [8]. Biofluids from NSCLC patients (including serum, urine, exhaled breath condensate, and sputum) have been found to harbor specific metabolic biomarkers with potential applications in clinical practice [10,11,12,13,14,15,16].

To date, the clinical application of metabolomics in general is limited due to several factors, including a lack of adequately controlled studies (i.e., incomparable patient populations) and a lack of standardized procedures for metabolite extraction, processing, and analysis [17]. Here, we applied metabolomics to serum and urine samples from a single patient population collected before and after surgical resection. We have applied two complementary and commonly used platforms to identify metabolites of interest: nuclear magnetic resonance (NMR) and liquid chromatography quadrupole time-of-flight mass spectrometry (LC-QTOF-MS). NMR can detect multiple metabolites in a short time with minimal sample preparation; LC-QTOF-MS is much more sensitive than NMR and can detect metabolites of very low concentrations [18,19]. We therefore present a prospective observational study in patients with known or suspected early-stage NSCLC and compare their metabolic profiles, screened by these two methods, from serum and urine collected before and after surgery. We hypothesized that the surgical resection of a malignant tumor affects the overall metabolome and causes significant changes in the concentrations of metabolites found in commonly analyzed biofluids. The main objective of this study was therefore to examine the overall metabolomic changes in serum and urine samples collected from patients with early-stage NSCLC before and after the surgical removal of the malignancy. This study opens a path to the development of non-invasive diagnostics, screening, and/or monitoring methods in managing NSCLC.

## 2. Materials and Methods

### 2.1. Patient Enrollment and Biofluid Sample Collection

Protocols were approved by the Research Ethics Board of the University of Manitoba, Canada (H2017:247). Each patient consented to participate in the study prior to accrual. Consecutive patients were screened for eligibility at a thoracic surgery clinic in Winnipeg, Manitoba, Canada. Eligible participants were adult patients (>18 years) with biopsy-proven or suspected stage I or II NSCLC (based on a maximum standardized uptake value on positron emission tomography or an increasing pulmonary tumor size in serial CT chest scans), and medically operable. Imaging data for each eligible patient were reviewed by a thoracic surgeon and a radiation oncologist. Clinical, imaging, surgical, and pathology data for enrolled patients were extracted from hospital paper charts and the Varian Medical Oncology application at Cancer Care Manitoba (CCMB) by a designated research assistant and verified by the principal investigator. All pre- and post-surgery samples were collected and prepared by the same research assistant, according to standardized protocols. Serum and midstream urine samples (8–10 mL) were collected within 4 weeks pre-surgery and within 4 months post-surgery. All samples were immediately stored at −80 °C and transferred to the laboratory for batched analysis by NMR and LC-QTOF-MS.

### 2.2. Nuclear Magnetic Resonance (NMR)

#### 2.2.1. Sample Preparation for NMR Analysis

The samples were thawed and prepared with the additives and parameters we have described previously [20]. The serum samples (300 μL) were mixed with a phosphate buffer (pH 7.4) containing 5 μM NaN_3_ at a ratio of 1:1 (*v*/*v*), followed by the addition of 20 μL of TSP (0.75%). The urine samples (400 μL) were mixed with 230 μL of a 0.2 M phosphate buffer (0.2% *w*/*v* NaN_3_) and 70 μL of Chenomx ISTD (IS-2, 5 mM DSS, 0.2% *w*/*v* NaN_3_). The samples were mixed by vortexing and centrifuged at 12,000× *g* at 4 °C for 5 min. A volume of 600 μL of each prepared sample was then transferred into a 5 mm NMR tube for analysis [20].

#### 2.2.2. NMR Analysis

The NMR experiments were conducted on a Bruker Ascend 600 Spectrometer operating at 600.27 MHz for proton nuclei and 150.938 MHz for carbon nuclei. Each sample was subject to two separate scans at a probe temperature of 298 K. A few randomly selected samples were run using the heteronuclear single quantum coherence (HSQC) method for additional analysis with a 65.5 k time domain, a 90° pulse width of 10 μs, a spectral width of 16 ppm, and a relaxation delay of 5 s. A total of 32 scans, with two dummy scans producing an acquisition time of 4.75 min, were acquired. Water was suppressed (at 2819 Hz, time domain 32.7 k) in all 1D Nuclear Overhauser Effect Spectroscopy (NOESY) runs. A total of 64 scans and 4 dummy scans were generated during the 7 min 45 s acquisition time. The 2D NMR spectra were achieved with a 90° pulse width of 10 μs and a relaxation delay of 1.5 s. The time-domain values for 2D NMR spectra were 2048 and 400 for F1 (16 ppm) and F2 (210 ppm), respectively. An acquisition time of approximately 11 h was needed to obtain 32 scans and 16 dummy scans. All NMR spectra were processed by MestReNova version 12.0.0–20080 as previously reported [20].

### 2.3. Liquid Chromatography Quadrupole Time-of-Flight Mass Spectrometry (LC-QTOF-MS)

#### 2.3.1. Sample Preparation for LC-QTOF-MS

The serum samples (100 μL) were spiked with 20 μL of Norvaline (0.03 mg/mL) as an internal standard and extracted with 200 μL of acetonitrile (ACN,). The urine samples (250 μL) were spiked with 10 μL of Norvaline (0.03 mg/mL) and extracted with 500 μL of ACN. The samples were then centrifuged at 12,000× *g* at 4 °C for 20 min. The supernatants of serum and urine were dried under a gentle stream of N_2_ and stored at −20 °C. The dried serum and urine samples were reconstituted with 100 μL of ddH_2_O:ACN (1:4) and 200 μL of ddH2O:ACN (4:1), respectively. Each sample was transferred into a glass insert in a gas chromatography (GC) vial for analysis [20].

#### 2.3.2. LC-QTOF-MS Analysis

The urinary and serum metabolites were separated using a 1260 Infinity HPLC system and were detected by a 6538 UHD Accurate Q-TOF MS system (Agilent Technologies, Santa Clara, CA, USA). The ionization of the separated metabolites was performed using an electrospray ionization source operating in positive (ESI+) and negative (ESI–) modes. The separation of the urinary metabolites was achieved using a 2.1 mm × 100 mm, 1.8 μm Zorbax SB-Aq column (Agilent Technologies) held at 45 °C and the serum metabolites were separated using a 2.1 mm × 50 mm, 1.8 μm Zorbax Extended-C18 column (Agilent Technologies) which was held at 55 °C. Water (ddH_2_O) and ACN with 0.1% formic acid were used as mobile phases using previously reported gradients [20]. The statistical analysis was carried out using Agilent MassHunter Acquisition software (B.07) and Mass Profiler Professional (MPP, 12.6.) as previously reported [20].

### 2.4. Statistical Analysis and Metabolite Identification

The fold change (FC) was calculated for each metabolite identified by dividing the larger of the pre- and post-surgery samples by the smaller value.

For the metabolites identified by NMR, the FCs between the identified classes of metabolites were compared using the Wilcoxon signed rank or the Kruskal–Wallis tests for the comparison of two or more classes, respectively. The chi-square and Fisher exact tests were used to compare the frequencies of regulatory direction. Within individuals, the pre- and post-surgery levels of metabolites were compared using the Wilcoxon signed rank test.

For LC-QTOF-MS, the MPP software (12.6.1) was used for the FC analysis by paired *t* tests (*p* < 0.05; ≥2-fold changes) with an asymptotic *p* value and the Benjamini–Hochberg multiple correction method was used to identify statistically significant metabolites.

The compounds identified through LC-QTOF-MS were further confirmed by mass spectra, retention time, and confidence scores against the Metlin database with >79,000 metabolites including 39,000 lipids and 168,000 peptides [20,21,22]. All the identified metabolites of significance were then re-classified based on the Human Metabolome Data Base (HMDB) [23].

## 3. Results

### 3.1. Patient Enrollment, Inclusion, and Exclusion

**Figure 1:**
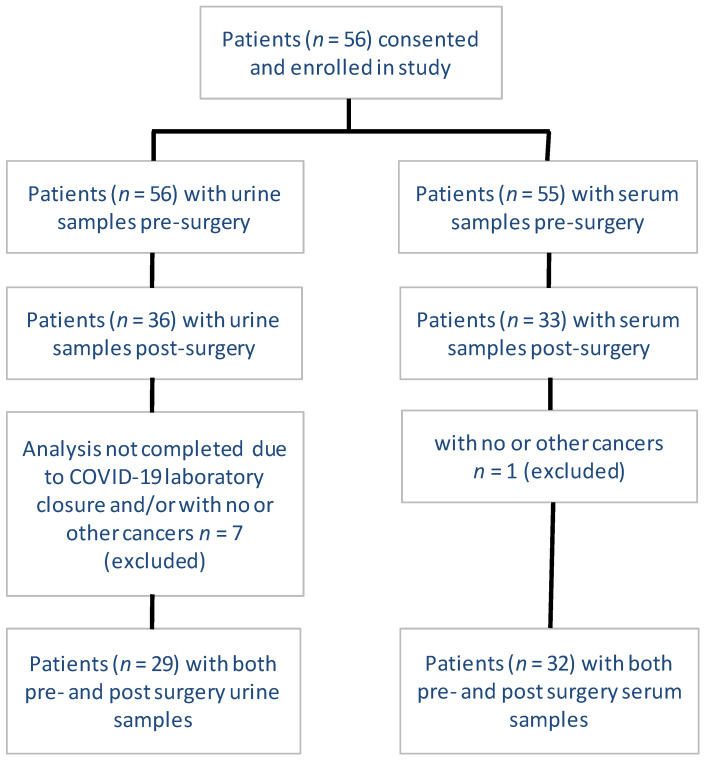
Patient enrollment and biofluid collection.

Between March 2018 and November 2019, 56 patients were enrolled in the study. Inclusion and exclusion criteria as well as details of the sample collection are presented in Figure 1. In total, 35 patients were eligible for analysis. From these individuals, NMR and QTOF analyses were completed on the urine samples from 29 patients and on the serum samples from 32 patients, collected both pre- and post-surgery. The mean time for serum and urine collection was 5 and 7 days before surgery and 56 and 57 days after surgery, respectively. Post-surgery, all but two patients had their biofluid samples taken before commencing any systemic therapy. A total of 86% of the patients were smokers or previous smokers. Each patient had a gross surgical resection of the tumor either through lobectomy/pneumonectomy (60%) or wedge resection/segmentectomy (40%). All patients had a confirmed pathological diagnosis of NSCLC, 80% had adenocarcinoma, 88% had no pathologically involved nodes (N0), and 12% had incidental intrapulmonary, hilar, or mediastinal (N1-N2) disease. ALK and PDL1 status was available in 66% of the patients. All patients had ALK negative tumors and a PDL1 tumor proportion score (TPS) of >50% was present in 17% of the tumors. EGFR status was not available. Mean (±SD) tumor size from pre-surgical chest CT scans was 2.4 ± 1.6 cm. Mean (±SD) tumor size from surgical pathology was 2.7 ± 1.9 cm. The mean maximum PET_SUV was 8.2 ± 6.5. The data are summarized in Table 1.

### 3.2. Inclusion, Exclusion, and Classification of Identified Metabolites

A total of 105 metabolites registering statistically significant differences pre- vs. post-surgery were identified by either LC-QTOF or NMR: LC-QTOF registered 71 metabolites of significance in the serum samples and 28 in the urine samples, and NMR registered 12 metabolites of significance in the serum samples and 37 in the urine samples. Of the metabolites identified by NMR, only one in the serum samples and five in the urine samples were included for analysis as those were statistically significant (*p* < 0.05). Each metabolite was reviewed based on the chemical formula, mass and atomic number, presence/absence, and the description of the compound in both HMDB [23] and the National Center for Biotechnology Information (NCBI) database [24]. From this analysis, 57 metabolites identified by LC-QTOF were judged to be either of exogenous origin or related to external substances (prescription drugs, food, or food additives) and were excluded from the analysis.

We therefore identified 48 metabolites of significance in the serum and urine samples and classified these based on their chemical composition and/or metabolic pathway per HMDB. Five classes of metabolites were identified: lipids and derivatives, proteins and derivatives, carboxylic acid and derivatives, carbohydrates, and purines/pyrimidines. The metabolites that did not fit into one of these classes were grouped as unclassified. A metabolite was labeled upregulated or downregulated if the levels increased or decreased after surgery, respectively, expressed as an absolute FC. Metabolites in each class with their respective atomic number/mass, polarity, FC, source biofluid (serum or urine) and the identifying platform (NMR/LC-QTOF) are presented in Table 2.

### 3.3. Pattern of Frequency and Distribution of the Identified Metabolites

The distribution and the frequency of the metabolites based on the biofluid, and class are presented in Table 3. There were 31 metabolites of significance in the serum samples and 17 in the urine samples that were not significantly different (*p* = 0.060). Lipids and their derivatives were the most frequently identified, followed by (in decreasing order of frequency) proteins and derivatives, carboxylic acid and derivatives, unclassified carbohydrates, and purines/pyrimidines.

### 3.4. Pattern of Dysregulation of the Identified Metabolites Post-Surgery

**Figure 2:**
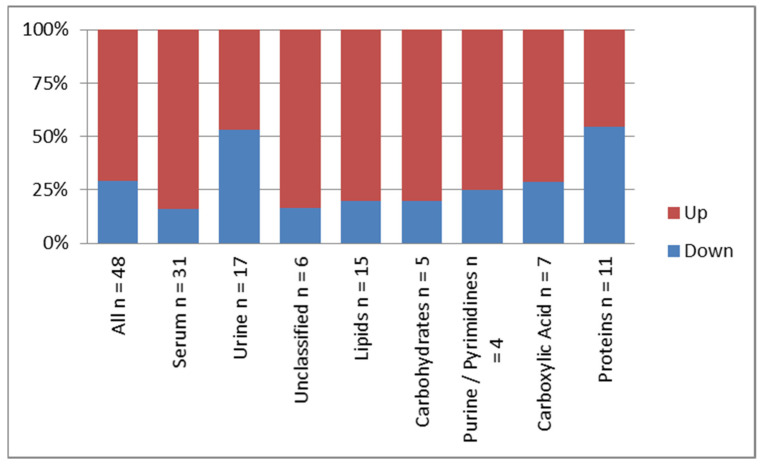
Pattern of regulation of identified metabolites based on biofluid and class of metabolites.

A higher proportion of metabolites were upregulated (*n* = 34) than were downregulated (*n* = 14) post-surgery (*p* = 0.0055). This ratio varied by biofluid: in serum, these proportions were 84% (26/31) upregulated and 16% (5/31) downregulated (*p* = 0.0002), and in urine, the proportions were similar at 46% (8/17) and 53% (9/17) up- and downregulated, respectively (*p* = 1). We found no significant differences in the proportion of the up- or downregulated metabolites based on chemical class (*p* = 0.48; see Figure 2).

### 3.5. Magnitude of Dysregulation of the Identified Metabolites Post-Surgery

**Figure 3:**
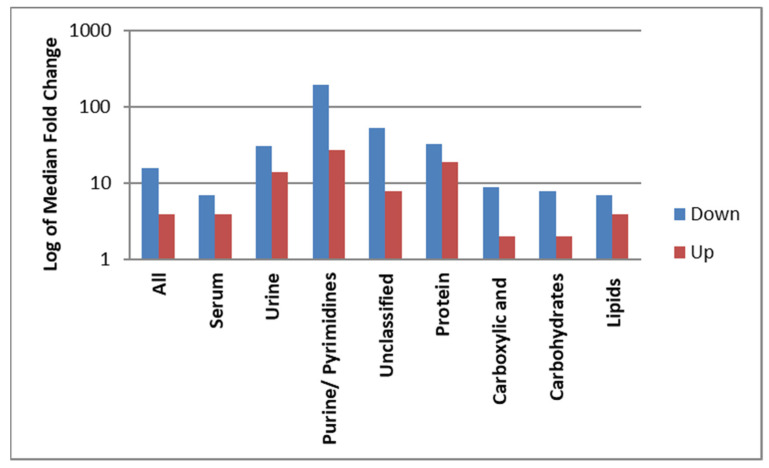
Magnitude of dysregulation. The magnitude of the downregulation of the metabolites after surgery was higher in both the serum and urine samples (*p* = 0.043); although this was not statistically significant, the difference was larger in the urine samples (*p* = 0.28) than the serum samples only (*p* = 0.69). The alteration in purines/pyrimidines (*p* = 0.045) and protein and derivatives (*p* = 0.027) was larger than those in the other classes of metabolites.

Median, minimum, and maximum changes in the levels of metabolites expressed as an absolute FC after surgery are presented in Table 4. The median FC was 4 and 16 for all up- and downregulated metabolites, respectively (*p* = 0.043). A similar pattern of dysregulation was observed when the data were grouped by biofluid but this was not significant: for the metabolites identified in the serum samples, the median FC (up- and downregulated) was 4 and 7, respectively (*p* = 0.69); for the metabolites identified in the urine samples, the median FC change was 14 and 31, respectively (*p* = 0.28). The maximum FC for upregulated metabolites in the serum and urine samples was 134 and 442, respectively. The maximum FC for downregulated metabolites in the serum and urine samples was 54 and 625, respectively. Purines/pyrimidines and proteins and derivatives had a larger dysregulation than those in the other classes of metabolites (*p* = 0.045 and *p* = 0.027, respectively). Purines/pyrimidines had the highest median FCs of 27 and 198, respectively, for up- and downregulated metabolites, followed by proteins and derivatives with median FCs of 19 and 33, respectively. The median FCs for lipids were 4 and 7 for up- and downregulated metabolites, respectively. Both carbohydrates and carboxylic acid and derivatives had a median FC of 2 for upregulated metabolites; for downregulated metabolites in these categories, the median FC was 8 for carbohydrates and 9 for carboxylic acid and derivatives. For the unclassified metabolites, the median FC was 8 and 54 for the upregulated and downregulated metabolites, respectively. These results are summarized in Figure 3.

### 3.6. Metabolites of Significance with >10 FC Dysregulation and Their Association with Pathogenesis of Cancer

There were 18 metabolites of significance dysregulated by >10-fold after surgery. Of these, seven were proteins and derivatives, three were purines, four were lipids, one was a carboxylic acid, one was an androgen, and two were unclassified (see Table 5). Six of these metabolites were directly associated with cancer pathogenesis based on the literature review. Leucyl proline, isopentenyladenine, fumaric acid, and asymmetric dime-thylarginine (ADMA) were identified in the urine samples and were downregulated by 625, 31, 17 and 16-fold, respectively, after surgery. N6-methyladenosine identified in the urine samples, and chenodeoxycholic/deoxycholic/glycoursodeoxycholic acid identified in the serum samples were upregulated with FCs of 27 and 28, respectively. Other metabolites of significance with FC > 100 included 3-methyl uric acid, N(alpha)-t-butoxycarbonyl-L-leucine, hypoglycin, and epinephrine without any previously direct association to cancer pathogenesis.

## 4. Discussion

### 4.1. Study Desgin and Metobolic Profile

We conducted an exploratory nontargeted metabolomics study for early-stage NSCLC patients eligible for a curative surgical resection. Two distinct aspects of our study design are (i) the patient population and (ii) the use of a radical treatment intervention to compare the metabolic profile of the same patient population, i.e., before and after the complete removal of a pulmonary cancerous lesion.

The metabolic profile obtained in this study represents a single patient population with small and localized tumors with no or minimum microscopic regional and no systemic metastasis. Surgical removal of the tumors provides a binary metabolic state in the *same* patient population, i.e., with and without a malignant tumor. Dysregulation of the metabolic profile was observed in the biofluids of these patients with intact pulmonary tumors, followed by a significant change in the profile after the surgical removal of the tumors. The proportion of the identified metabolites in the serum samples (65%) and the urine samples (35%) was not statistically different. However, the pattern of the dysregulation of the metabolites after surgery was distinct. A higher proportion of metabolites increased in concentration after surgery in the serum and urine samples, and in the serum samples only compared to the urine samples. In contrast, the proportion of urinary metabolites that were increased or decreased after surgery was similar. The allocated chemical class of metabolite did not influence the proportion of metabolites following a particular trajectory of dysregulation after surgery (Figure 2). The magnitude of the downregulation of the metabolites after surgery was higher in both the serum and urine samples, although this was not statistically significant; the difference was larger in the urine samples. (Table 4 and Figure 3).

### 4.2. Lipids and Derivatives

Among the identified classes of metabolites, lipids were the most frequently identified and included fatty acids, bile acids, carnitines, phosphatidylglycerol, phosphatidylinositol, prostaglandins, and products of the mevalonate pathway. All lipid metabolites except one were identified in the serum samples. Most lipid metabolites (80%) were upregulated after surgery with a moderate change in their profile (median FC = 4). Isopentenyladenine, the only lipid identified in the urine samples linked to the mevalonate pathway, was downregulated (median FC = 31). Based on our review of the literature, the most common lipid metabolic alterations linked to carcinogenesis are in fatty acid metabolism, arachidonic acid metabolism, cholesterol metabolism, and peroxisome proliferator-activated receptors (PPAR) signaling [25]. PPAR plays a significant role in chronic inflammatory conditions leading to cancer development [26]. The uptake, activation, and synthesis of fatty acids by tumors is facilitated through enzymes such as acetyl coenzyme A, and fatty acid synthase (FASN) [27,28]. The upregulation of FAS in early-stage lung cancer tumors has been associated with aggressive clinical behavior and a poor prognosis [29,30]. The key enzyme stearoyl coenzyme A desaturase 1 (SCD1), involved in the formation of palmitoleic and oleic acids has been implicated in adenocarcinomas of the lung in tumor initiation and invasion and is a potential target for therapeutic intervention [31]. Members of the adenosine triphosphate-binding cassette (ABC) family of proteins have been correlated with a poor response to platinum-based chemotherapy in NSLC [32]. ATP citrate lyase (ACLY), which is involved in fatty acid synthesis, has been associated with tumorigenesis and is a potential prognostic biomarker [28,33]. In the current study, 2-propylpent-3-enoic acid, a fatty acid, was upregulated in the serum samples by 50-fold post-surgery. We could not find any previous association of this metabolite to the pathogenesis of cancer. Isopentenyladenine, a product of the mevalonate pathway, decreased by 31-fold in the urine samples after surgery. The mevalonate pathway is frequently overactive in cancer cells [34] and regulates cholesterol synthesis and the formation of 3-hydroxy-3-methylgrutaryl coenzyme A (HMG-CoA). HMG-CoA is reduced to mevalonate by HMG-CoA reductase (HMGCR); HMGCR is a potential therapeutic target for cholesterol lowering drugs (such as statins) to treat cancer [34,35,36,37]. Chenodeoxycholic/deoxycholic/glycoursodeoxycholic acid, a bile acid, was upregulated by 28-fold in serum post-surgery. Bile acids have been implicated in the pathogenesis of several malignancies including colorectal, breast, hepatocellular, and renal cancers [38,39].

### 4.3. Proteins and Derivatives

Of the metabolites we identified, 23% were related to protein metabolism, with almost similar frequencies of up- and downregulation. However, as a class of metabolites, the overall magnitude of the alteration and the decrease in the metabolite levels after surgery was significant (Table 4, Figure 3). Cancer cells proliferate and survive by upregulating the synthesis of essential and nonessential amino acids, facilitated by specific transport systems, stromal cells, gene silencing, and redox homeostasis. Various cell signaling pathways are altered to generate nucleotides, reactive oxygen species (ROS), scavenging molecules, and oncometabolites to favor the proliferation of cancer cells [40,41,42]. We found that two metabolites identified in the urine samples, leucyl proline (Pro Leu) and ADMA, which have previously been associated with cancer pathogenesis, profoundly decreased after surgery (Table 5). Pro Leu, a dipeptide composed of L-leucine and L-proline, was downregulated by 625-fold in urine. Recent reviews have highlighted a clinically significant role for proline in cancer metabolism [43]. Through a series of reactions, proline metabolism is initiated by converting proline into pyrroline-5-carboxylate, facilitated by the enzyme proline dehydrogenase/proline oxidase (PRODH/POX). High levels of proline result in the upregulation of PRODH, which in turn results in the production of ROS. PRODH is induced by p53 and can be up- or downregulated depending upon the type of the cancer and cell environment [44]. ADMA, an amino acid, decreased by 16-fold in urine post-surgery. Higher levels of this metabolite have been found in lung cancer patients [45]. The dipeptide aspartyl glycine was downregulated by 50-fold in urine. Pre-clinical studies using cancer cell lines have indicated that glycine consumption and the mitochondrial glycine biosynthetic pathway are associated with cancer cell proliferation [46]. Glutamic acid n-butyl ester was upregulated in urine by 65-fold. Glutamic acid (glutamate) is a proteinogenic nonessential amino acid and a bioenergetic substrate for proliferating malignant cells. It is involved in tumor development as a growth factor and a signal mediator facilitated through metabotropic glutamate receptors and ionotropic glutamate receptors found in cancerous tumors [47].

### 4.4. Purines and Pyrimidines

Purines are the most commonly found metabolites in normal cells. They are the primary building blocks in DNA and RNA synthesis and provide the energy and cofactors needed for normal and essential cellular functions [48]. Altered purine metabolism in cancer cells is a component of proliferation, tumor immune response, invasiveness, and metastasis and has led to novel therapeutic interventions in the field of oncology such as the antipurine metabolite drugs 6-mercaptopurine, 6-thioguanine, and methotrexate [49]. In this study, the magnitude of the alteration of purines after surgery was significant (Table 4 and Figure 3). Noticeably, N6-methyladenosine was identified in urine, and was upregulated by 27-fold post-surgery. N6-methyladenosine is an endogenous methylated adenine produced by the degradation of transfer ribonucleic acid (tRNA) and found in urine [23]. This is the most commonly identified post-transcriptional modification of mRNA and is primarily a reversible process facilitated by the enzymes methyltransferase and demethylase. Recent research has revealed a critical role for N6-methyladenosine in the oncogene regulation and pathogenesis of several human cancers, including NSCLC [50,51,52,53]. Methyl uric acid, a xanthine and a purine derivative, was downregulated by 198-fold. Uric acid metabolism may have a potential role in carcinogenesis through its role both as an anti and pro oxidant [54]. In a small pilot study involving breast cancer patients, urinary methyl uric acid identified by LC-QTOF was reduced by 0.6-fold in urine compared to the patients with no cancer [55].

### 4.5. Carboxylic Acid Derivatives and Carbohydrates

There were seven metabolites identified as carboxylic acid or derivatives. Fumaric acid was found to be decreased by 17-fold after surgery and was detected in the urine samples. This is one of compounds of the Krebs cycle and a biproduct of succinic acid oxidation. It has been found to be significantly altered in the serum of lung cancer patients compared to healthy individuals [56,57].

The altered glucose metabolism in cancer cells has been known for the past 100 years and is based on the “Warburg effect”, which is related to the increased uptake and utilization of glucose with disproportionately increased lactate production by proliferating cancer cells in the presence of sufficient oxygen or hypoxic conditions [58]. In our study, all identified metabolites related to carbohydrates were upregulated with a relatively minimal change (median FC = 2), except beta-cortol, which was downregulated by 8-fold and found in the serum samples. Beta-cortol belongs to a group of cortisol metabolites that are O-glycosyl compounds, in which a sugar is bonded through one carbon to another group via a O-glycosidic bond [36]. An altered cortisol metabolism has been demonstrated in advanced cancers [59]. There is evidence that microcellular lung cancer produces ACTH (and hence increased cortisol levels), which could serve as potential biomarker for the early diagnosis of lung cancer [60].

### 4.6. Endocrine Factors

The androgens androstanediol and androstenedione were identified in the serum samples. While androstenedione was modestly elevated (FC = 8), androstanediol was downregulated by 54-fold. Androgens may have a role in the pathophysiology of lung cancer as androgen receptors have been found in lung cancer tumors. A retrospective study of NSCLC patients by Harlos et al. [61] indicated that androgen pathway manipulation was prognostic and associated with improved survival in lung cancer patients who received 5-alpha reductase inhibitors. Dopamine and epinephrine are catecholamines. Both were upregulated after surgery and identified in the serum samples. While dopamine only increased by 5-fold, epinephrine increased by 134-fold post-surgery. These compounds have opposite roles in tumor angiogenesis. Dopamine and epinephrine inhibit and promote tumor angiogenesis, respectively [62]. Tetrahydrobiopterin/sapropterin (BH4, THB) is a biopterin that was upregulated post-surgery by 8-fold. Biopterin may have a role in tumor progression [63]. N-desmethylaminopyrine, a phenyl pyrazole, increased by 10-fold in serum. We could not find any previously known association of this compound to carcinogenesis.

A summary of the metabolites of significance in this study and the associated metabolic pathways employed in carcinogenesis is presented in Table 6.

### 4.7. Strengths and Limitations of the Study

This study is limited by the small sample size and the large number of metabolites which were assessed. These two factors render this study underpowered for any meaningful multivariable analysis to determine the effect of clinical and pathological variables on the metabolic profile of this cohort. Nonetheless, this study is designed to address the effect of treatment intervention (i.e., surgical tumor extraction) on the patient’s metabolic profile. The pre- and post-exposure design set up each patient to serve as their own control, thus eliminating any bias introduced by comparisons *between* individuals. Furthermore, surgical resection in early-stage cancer patients achieves a more complete eradication of cancer compared to radiation and/or systemic (chemo) therapy. Surgery thus represents a unique intervention to compare two metabolic states in the same patient population, which may be considered “with” and “without” cancer.

## 5. Conclusions

We have conducted a prospective observational study in a single cohort of early-stage NSCLC patients to compare biofluid metabolomics before and after the surgical removal of pulmonary tumors. We detected a dysregulation of the primary metabolic pathways in urine and serum samples by both NMR and LC-QTOF-MS, evidenced by the changes in 31 and 17 metabolites in the serum and urine samples, respectively, obtained from 35 patients. There was no significant difference in the distribution of metabolites between the serum and urine samples, but the pattern of dysregulation was different. More metabolites were upregulated in the serum samples with a consistent pattern of dysregulation in the urinary metabolites after surgery. The chemical class of the metabolites did not influence the proportion of metabolites that were up- or downregulated. The magnitude of downregulation after surgery was higher than that of upregulation in both the serum and urine samples, and although not statistically significant, this difference was larger in the urine samples. Alteration in purines/pyrimidines and proteins and derivatives was larger than that of other classes of metabolites detected in our study, suggesting the importance of these as potential biomarkers for screening, diagnostics, or monitoring. Based on the magnitude of alteration and the previously known association to NSCLC and/or any other cancer, we identified six metabolites of significance: in both the serum and urine samples, leucyl proline, ADMA, isopentenyladenine, and fumaric acid were downregulated after surgery; N6-methyladenosine and deoxycholic acid moieties were upregulated. Four other metabolites with a significant dysregulation, FC > 100 after surgery, included 3-methyl uric acid, N(alpha)-t-butoxycarbonyl-L-leucine, hypoglycin, and epinephrine; there is no known direct association of these metabolites to cancer pathogenesis, and this is a new observation. Further studies with larger cohorts that include patients with advanced-stage lung cancer are warranted to expand on our findings and progress toward the clinical application of metabolomics in cancer diagnosis and treatment.

## Figures and Tables

**Table 1:** Clinical characteristics of the patient cohort.

Characteristics	Total (*n* = 35)	Urine (*n* = 29)	Serum (*n* = 32)
Age Mean (SD) in years	64.7 (7.4) (*n* = 34)	63.8 (7.0) (*n* = 28)	64.6 (7.5) (*n* = 31)
Females	63% (22)	62% (18)	62% (20)
Males	37% (13)	38% (11)	38% (12)
Smoker	34% (10)	36% (10)	35% (9)
Ex-Smoker	52% (15)	50% (14)	50% (13)
Never Smoked	14% (4)	14% (4)	15% (4)
Diabetes	17% (6)	10% (3)	19% (6)
COPD	49% (17)	48% (14)	50% (16)
Previous cancers	29% (10)	31% (9)	28% (9)
On steroids oral/inhalers	0%	0%	0%
Squamous cell carcinoma	14% (5)	14% (4)	16% (5)
Adenocarcinoma	80% (28)	79% (23)	81% (26)
Other	6% (2)	7% (2)	3% (1)
Right Upper Lobe	29% (10)	31% (9)	25% (8)
Right Lower Lobe	20% (7)	17% (5)	22% (7)
Left Upper Lobe	31% (11)	34% (10)	31% (10)
Left Lower Lobe	19% (7)	17% (5)	22% (7)
PET before surgery	86% (30)	86% (25)	84% (27)
*Type of surgery:*			
Wedge Resection/Segmentectomy	40% (14)	38% (11)	41% (13)
Lobectomy	51% (18)	55% (16)	50% (16)
Pneumonectomy	3% (1)	3% (1)	3% (1)
Wedge and Lobectomy	6% (2)	3% (1)	6% (2)
Pathological Stage (*n* = 34)		*n* = 28	*n* = 31
T1-T2, N0 M0	79% (27)	75% (21)	77% (24)
T3-T4, N0 M0	9% (3)	11% (3)	10% (3)
T1-T4, N1-2 M0	12% (4)	14% (4)	13% (4)
Mean Tumor size based on CT scan before surgical resection Mean (SD) in cm	2.4 (1.6) (*n* = 35)	2.5 (1.7) (*n* = 29)	2.5 (1.7) (*n* = 32)
Mean Tumor size base on surgical pathology Mean (SD) in cm	2.7 (1.9) (*n* = 34)	2.8 (2.0) (*n* = 28)	2.8 (1.9) (*n* = 31)
Mean Maximum PET_SUV Mean (SD)	8.2 (6.5) (*n* = 30)	8.2 (6.9) (*n* = 25)	8.1 (6.7) (*n* = 27)
PDL1: <1% (10/23)	43%	47% (9/19)	45% (10/22)
PDL1: 1–49% (9/23)	39%	42% (8/19)	41% (9/22)
PDL1: >50% (4/23)	17%	11% (2/19)	14% (3/22)
ALK: Negative (*n* = 23)	100%	100% (*n* = 20)	100% (*n* = 22)

Notes: TNM Staging per AJCC, TNM 6th edition. COPD, chronic obstructive airway disease; SD, standard deviation.

**Table 2:** Classification of altered metabolites of significance.

Compound	Formula	m/z	Polarity	FC	*p* Value	Reg	Class	Biofluid	Platform
**Lipids and Derivatives**									
2-Propylpent-3-enoic acid	C_8_H_14_O_2_	160.1328	+	50	<0.0001	up	Fatty Acid	Serum	QTOF
13,14-Dihydro PGE1/Prostaglandin F1a	C_20_H_36_O_5_	713.493	+	5	0.047	up	Prostaglandins	Serum	QTOF
2-Hexenoylcarnitine	C_13_H_23_NO_4_	258.1654	+	16	0.013	up	Acyl Carnitines	Serum	QTOF
2-Octenoylcarnitine	C_15_H_27_NO_4_	286.2013	+	3	0.026	up	Acyl Carnitines	Serum	QTOF
Chenodeoxycholic/ Deoxycholic acid glycine conjugate/ Glycoursodeoxycholic acid	C_26_H_43_NO_5_	450.3208	+	28	0.0005	up	Bile Acid	Serum	QTOF
Cholic acid	C_24_H_40_O_5_	426.3246 ^^^	+	4	0.026	down	Bile Acid	Serum	QTOF
cis-5-Tetradecenoylcarnitine	C_21_H_39_NO_4_	370.2948	+	4	0.033	up	Acyl Carnitines	Serum	QTOF
Decanoylcarnitine	C_17_H_33_NO_4_	3,162,481	+	2	0.0001	up	Acyl Carnitines	Serum	QTOF
Dodecanoylcarnitine	C_19_H_38_NO_4_	344.2792	+	6	0.0009	up	Acyl Carnitines	Serum	QTOF
Isopentenyladenine	C_16_H_23_N_5_O_5_	204.1242	+	31	0.0001	down	Mevalonate Pathway	Urine	QTOF
L-Carnitine	C_7_H_15_NO_3_	162.1124	+	3	<0.0001	up	Carnitines (Lipid Metabolism)	Serum	QTOF
L-Octanoylcarnitine	C_15_H_29_NO_4_	288.2166	+	2	0.0008	up	acyl carnitines	Serum	QTOF
LysoPC(P-18:1)	C_26_H_52_NO_7_P	522.3552	+	3	0.026	up	Fatty Acid	Serum	QTOF
PG(18:1/18:2)	C_42_H_77_O_10_P	773.536	+	6	0.0087	up	phosphatidyl glycerols	Serum	QTOF
PI(16:0/18:1)	C_43_H_81_O_13_P	854.5691 ^^^	+	7	0.0002	down	phosphatidy linositols	Serum	QTOF
**Proteins and Derivatives**									
4-Guanidinobutanoic acid	C_5_H_11_N_3_O_2_	163.1156 ^^^	+	4	0.029	up	amino acid (Gamma)	Serum	QTOF
Aspartyl glycine	C_8_H_13_N_3_O_6_	248.0938	+	50	0.0006	down	dipeptide	Urine	QTOF
Asymmetric dimethylarginine (ADMA)	C_8_H_18_N_4_O_2_	203.1505	+	16	0.044	down	amino acid	Urine	QTOF
Hypoglycin	C_7_H_11_NO_2_	142.0875	+	442	<0.0001	up	amino acid	Urine	QTOF
Isodesmosine	C_24_H_40_N_5_O_8_	527.296	+	19	0.043	up	amino acid	Serum	QTOF
L-Glutamic acid n-butyl ester	C_9_H_17_NO_4_	204.1233	+	65	0.0009	up	amino acid	Urine	QTOF
L-Isoleucyl-L-proline	C_11_H_20_N_2_O_3_	229.152	+	2	0.0005	up	dipeptide	Serum	QTOF
N(alpha)-t-Butoxycarbonyl-L-leucine	C_11_H_21_NO_4_	232.1547	+	161	<0.0001	down	amino acid	Urine	QTOF
Pro Leu	C_11_H_20_N_2_O_3_	229.1548	+	625	<0.0001	down	dipeptide	Urine	QTOF
Serine	C_3_H_7_NO_3_			1	0.030	down	amino acid	Serum	NMR
**Carbohydrates**									
Myoinositol	C_6_H_12_O_6_	203.0524 *	–	2	0.0002	up	Carbohydrate	Serum	QTOF
Glyceraldehyde	C_3_H_6_O_3_	203.0524 *	–	2	0.0001	up	Carbohydrate	Serum	QTOF
Glucose	C_6_H_12_O_6_			2	0.0499	up	Carbohydrate	Urine	NMR
Lactate	C_3_H_5_O_3_	203.0524 *	–	2	0.013	up	Glycolysis Product	Urine	NMR
Beta-Cortol				8	0.013	down	Carbohydrate	Serum	QTOF
**Purine/Pyrimidines**									
1-Methyladenine	C_6_H_7_N_5_	321.1307 *	+	59	<0.0001	up	Purine	Urine	QTOF
3-Methyluric acid	C_6_H_6_N_4_O_3_	183.0515	+	198	<0.0001	down	Purine	Urine	QTOF
5-Acetylamino-6-formylamino-3-methyluracil	C_8_H_10_N_4_O_4_	249.0608 *	+	3	0.029	up	Hydroxypyrimidine	Serum	QTOF
N6-Methyladenosine	C_11_H_15_N_5_O_4_	282.1199	+	27	0.0085	up	purine nucleoside	Urine	QTOF
**Carboxylic acid and Derivatives**									
cis-Aconitate	C_6_H_6_O_6_			1	0.035	up	carboxylic acid	Urine	NMR
Malonate	C_3_H_3_O_4_			2	0.014	up	carboxylic acid	Urine	NMR
4-Hydroxycyclohexylcarboxylic acid	C_7_H_12_O_3_	162.1126 ^^^	+	3	<0.0001	up	carboxylic acid	Serum	QTOF
Fumaric acid	C_4_H_4_O_4_	139.0026	+	17	0.011	down	carboxylic acid	Urine	QTOF
Guanidinosuccinic acid	C_5_H_9_N_3_O_4_	176.0654	+	3	0.023	up	carboxylic acid (aspartic acid)	Serum	QTOF
Proline betaine	C_7_H_14_NO_2_	144.1014	+	2	0.024	up	carboxylic acid (proline derivative)	Serum	QTOF
Succinate	C_4_H_6_O_4_			2	0.046	down	carboxylic acid	Urine	NMR
**Unclassified**									
Androstanediol	C_19_H_32_O_2_	623.4382 ^$^	+	54	<0.0001	down	Androgens	Serum	QTOF
Dopamine	C_8_H_11_NO_2_	154.0823	+	5	<0.0001	up	catecholamine	Serum	QTOF
Epinephrine	C_9_H_13_NO_3_	184.0944	+	134	<0.0001	up	catecholamine	Serum	QTOF
Androstenedione	C_19_H_26_O_2_	287.2041	+	8	0.023	up	Androgens	Serum	QTOF
Tetrahydrobiopterin/Sapropterin (BH4, THB)	C_9_H_15_N_5_O_3_	500.2767 ǂ	+	8	0.0031	up	Biopterin	Serum	QTOF
N-Desmethylaminopyrine	C_12_H_15_N_3_O	218.1378	+	10	0.0003	up	phenylpyrazoles	Serum	QTOF

Notes: * Na adduct; ^ NH_4_^+^ adduct; ^$^ [2M + K]^+^; ǂ [2M + NH_4_]^+^.

**Table 3:** Categorization of the metabolites based on biofluid and their chemical class.

Source or Class of Metabolites	Frequency *n* (%)
All	48 (100)
Serum	31 (65)
Urine	17 (35)
Lipids and Derivatives	15 (31)
Protein and Derivatives	11(23)
Carboxylic Acid and Derivatives	7 (15)
Unclassified	6 (13)
Carbohydrates	5 (10)
Purine/Pyrimidines	4 (8)

**Table 4:** Fold change (FC) in metabolites post-surgery based on biofluid and chemical class.

Class of Metabolites	Upregulated				Downregulated				*p* Value
	N	Med FC	Min FC	Max FC	*n*	Med FC	Min FC	Max FC	
All	34	4	1	442	14	16	1	625	0.043
Biofluid									
Serum	26	4	2	134	5	7	1	54	0.69
Urine	8	14	1	442	9	31	2	625	0.28
Class									
Lipids and derivatives	12	4	2	50	3	7	4	31	
Proteins and derivatives	5	19	2	442	6	33	1	625	
Carbohydrates	4	2	2	2	1	8			
Purine/Pyrimidines	3	27	3	59	1	198			
Carboxylic acid and derivatives	5	2	1	3	2	9	2	17	
Unclassified	5	8	5	134	1	54			

Notes: *Upregulated* indicates that the metabolite level increased post-surgery. *Downregulated* indicates that the metabolite level decreased post-surgery. *n*, number of metabolites; Max, maximum; Med, median; Min, minimum.

**Table 5:** Metabolites of significance (FC > 10).

Class	Biofluid	Metabolite	Regulation	Fold Change	Previously Known Association to Cancer Pathogenesis
Protein and Derivatives	Urine	**Pro Leu**	down	625	**Yes**
Purine/Pyrimidines	Urine	3-Methyluric acid	down	198	Yes
Protein and Derivatives	Urine	N(alpha)-t-Butoxycarbonyl-L-leucine	down	161	No
Androgens	Serum	Androstanediol	down	54	Yes
Protein and Derivatives	Urine	Aspartyl glycine	down	50	Yes
Lipid and Derivatives	Urine	**Isopentenyladenine**	down	31	**Yes**
Carboxylic Acid and Derivatives	Urine	**Fumaric acid**	down	17	**Yes**
Protein and Derivatives	Urine	**Asymmetric dimethylarginine (ADMA)**	down	16	**Yes**
Protein and Derivatives	Urine	Hypoglycin	up	442	No
Unclassified	Serum	Epinephrine	up	134	Yes
Protein and Derivatives	Urine	L-Glutamic acid n-butyl ester	up	65	Yes
Purine/Pyrimidines	Urine	1-Methyladenine	up	59	No
Lipid and Derivatives	Serum	2-Propylpent-3-enoic acid	up	50	No
Lipid and Derivative	Serum	**Chenodeoxycholic/Deoxycholic/** **Glycoursodeoxycholic acid**	up	28	**Yes**
Purine/Pyrimidines	Urine	**N6-Methyladenosine**	up	27	**Yes**
Protein and Derivative	Serum	Isodesmosine	up	19	No
Lipid and Derivative	Serum	2-Hexenoylcarnitine	up	16	Possible
Unclassified	Serum	N-Desmethylaminopyrine	up	10.3	No

Notes: Based on the magnitude of alteration of the metabolites (>10 fold) after surgery and previously known association to NSCLC and or any other cancer, six metabolites of significance have been identified marked **in bold.**

**Table 6:** Metabolites of significance and the associated metabolic pathways.

Metabolite	Regulation	Metabolic Pathway
Pro Leu	down	Proline metabolism facilitated by PRODH/POX
3-Methyluric acid	down	Purine Metabolism
Androstanediol	down	Androgen pathway through AR in NSCLC
Aspartyl glycine	down	Mitochondrial glycine biosynthetic pathway
Isopentenyladenine	down	Mevalonate pathway
Fumaric acid	down	Krebs cycle
Asymmetric dimethylarginine (ADMA)	down	Overexpression of protein arginine methyl transferase
Epinephrine	up	Angiogenesis
2-Propylpent-3-enoic acid	up	Fatty acid metabolism
L-Glutamic acid n-butyl ester	up	Growth factor, metabotropic/ionotropic glutamate receptors
Chenodeoxycholic/Deoxycholic/ Glycoursodeoxycholic acid	up	Over expressed FXR
N6-Methyladenosine	up	Degradation of tRNA

Notes: AR, androgen receptors; FXR, farnesoid X receptor; NSCLC, non–small cell lung cancer; PRODH/POX, proline dehydrogenase/proline oxidase; tRNA, transfer ribonucleic acid.

## Data Availability

Metabolite data from NMR and LC-QTOF-MS, not reported in Table 2 is available on: https://doi.org/10.6084/m9.figshare.14781396.v1.
